# Emergency medical care of patients with psychiatric disorders -  challenges and opportunities: Results of a multicenter survey

**DOI:** 10.1186/s12873-022-00722-5

**Published:** 2022-10-28

**Authors:** Benedikt Schick, Benjamin Mayer, Markus Jäger, Bettina Jungwirth, Eberhard Barth, Martin Eble, Christoph Sponholz, Claus-Martin Muth, Carlos Schönfeldt-Lecuona

**Affiliations:** 1 Department of Anesthesiology and Intensive Care Medicine, University Hospital Ulm, Albert-Einstein-Allee 23, 89081 Ulm, Germany; 2 Institute of Epidemiology and Medical Biometry, Ulm University, Schwabstraße 13, 89075 Ulm, Germany; 3 Department of Psychiatry, Psychotherapy and Psychosomatic, District Hospital Kempten, Kempten, Germany; 4 Department of Anesthesiology, Intensive Care Medicine, Emergency Medicine & Pain Therapy, Klinikum Friedrichshafen GmbH, Röntgenstraße 2, 88048 Friedrichshafen, Germany; 5 Department of Anesthesiology and Intensive Care Medicine, Jena University Hospital, Friedrich Schiller University Jena, Am Klinikum 1, 07747 Jena, Germany; 6 Department of Psychiatry and Psychotherapy III, University Hospital Ulm, Leimgrubenweg 12–14, 89075 Ulm, Germany

**Keywords:** Emergency medicine, Emergency therapy, Mental disorders, Primary health care

## Abstract

**Background:**

Pre-clinical psychiatric emergencies are generally treated by emergency medical staff. The subsequent clinical treatment is often conditioned by interaction problems between emergency medical staff and psychiatric clinical staff.

**Objectives:**

To identify problems affecting interaction between emergency medical and psychiatric care of mentally ill patients and pinpoint aspects of optimized emergency care.

**Methods:**

To shed light on the interaction problems an anonymous, questionnaire-based, nonrepresentative survey of 98 emergency physicians (EM) and 104 psychiatrists (PS) practicing in acute psychiatry was conducted between March 1, 2021 and October 1, 2021.

**Results:**

The chi-square test for multiple response sets revealed consistently significant differences (p < 0.001) between EM and PS with respect to the questions analyzed. Approximately 36% of EM reported not to be adequately qualified to handle psychiatric emergencies (p = 0.0001), while around 50% of respondents were neutral in their assessment in how to deal with psychiatric emergencies. 80% of EM reported a negative interaction (rejection of patients) with PS when referring a psychiatric emergency patient to the acute psychiatric unit. The most common reasons for refusal were intoxication (EM: 78.8%, PS: 88.2%), emergency physician therapy (EM: 53.8%, PS: 63.5%), and not resident in the catchment area of the hospital (EM 68.8%, PS: 48.2%). In the casuistry presented, most respondents would choose “talk down” for de-escalation (EM: 92.1%, PS: 91.3%). With respect to drug therapy, benzodiazepine is the drug of choice (EM: 70.4%, PS: 78.8%). More EM would choose an intravenously (i.v.) or a Mucosal Atomization Device (MAD) administration as an alternative to oral medication (i.v.: EM: 38.8%, PS: 3.8%, p = 0.001, MAD: EM: 36.7%, PS: 10.6%, p = 0.006). Significantly more EM would seek phone contact with the acute psychiatric hospital (EM: 84.7%, PS: 52.9%, p = 0.0107). A psychiatric emergency plan was considered useful in this context by more than 90% of respondents. The need for further training for EM with regard to treating psychiatric clinical syndromes was considered important by all respondents. In particular, the topics of “psychogenic seizure,“ “intoxication,“ and “legal aspects of psychiatric emergencies” were considered important (Mann-Whitney U test, p < 0.001).

**Conclusion:**

The interaction-related problems identified in the emergency medical care of pre-clinical psychiatric patients relate to non-modifiable, structural problems, such as insufficient admission capacity and non-existent or inadequate monitoring capabilities in acute psychiatric hospitals. However, factors such as the education and training of EM and communication between EM and PS can be improved. Developing personalized emergency care plans for psychiatric patients could help to optimize their care.

**Supplementary Information:**

The online version contains supplementary material available at 10.1186/s12873-022-00722-5.

## Background

According to the World Health Organization (WHO), there has been a 13% increase in mental disorders worldwide over the past decade (as of 2017) [1]. For example, about 5% of all people worldwide suffer from depression [2]. These developments inevitably impact emergency medicine [3, 4]. Thus, about 500,000 emergency medical interventions with psychiatric indications are reported annually for the Federal Republic of Germany, and this figure is increasing [5]. In addition, psychiatric patients are significantly more likely to use emergency departments than somatic patients [6, 7]. Concomitant comorbidities can complicate the pre-clinical care process [8–13].

Although emergencies with psychiatric indications are about as common as neurological or traumatological emergencies in Germany, many emergency physicians (EM) do not feel adequately qualified to treat them [9–11, 14]. Indeed, psychiatric emergencies differ significantly from other emergencies in terms of the predominantly syndromal classification of disease symptoms, lack of algorithms, and ultimately the individual experience of the emergency physician [9, 15–17].

The real dilemma with respect to the pre-clinical care of psychiatric emergency patients, however, is the fact that psychiatric hospitals not always guarantee the admission of an emergency patient for various reasons. In addition to infrastructural concerns, such as limited admission capacity, practical considerations such as the lack of monitoring capabilities in the event of severe intoxication often impede the admission of the psychiatric emergency patient. Nevertheless, there is a lack of reliable data from emergency and rescue medicine on the admission of psychiatric patients to such facilities. While simply inconceivable for patients experiencing acute myocardial infarction or polytrauma, admission to an unsuitable hospital is often the reality for the mentally ill patient, who is frequently finally admitted to a central Emergency Department as alternative, due to rejection in psychiatric units. Therefore, the aim of the present study was to identify problems affecting interaction between emergency medical and psychiatric care facilities treating mentally ill patients by surveying EM and psychiatrists (PS) and to inquire about aspects that characterize optimized emergency care.

## Study design and methods

### Study design

An anonymized, questionnaire-based, non-representative survey of EM at five maximum care hospitals and one primary care hospital was conducted between March 01, 2021 and October 01, 2021. EM responded to questionnaires that specifically addressed the problems and problem-solving strategies involved in providing emergency medical care to psychiatric patients. The psychiatric questionnaires were answered by PS at five university hospitals and seven peripheral psychiatric hospitals. These questionnaires were designed to require PS to partially assume the role of EM. In particular, the questions addressed to EM were modified so that the PS could contribute their expert knowledge to solve the problems concerned. The study was designed in the Department of Anesthesiology and Intensive Care Medicine and the Department of Psychiatry and Psychotherapy III at Ulm University Hospital. The local ethic review board (ethics committee of the University of Ulm, Germany) approved the study procedures. The anonymity of the questionnaire and thus the omission of the requirement for a separate data protection statement was confirmed by the Center for Clinical Studies at the University of Ulm.

### Characteristics of the questionnaires

The questionnaires were designed by an anesthetist practicing emergency medicine and an experienced psychiatrist. Respondents completing the questionnaire were given the option to supplement their answers with free text. After an internal pretest and subsequent appropriate adjustment (validation part), the questionnaire was released for use in the medical centers mentioned above. The questionnaires had three domains (see supplement): (i) in the first part, questions were related to respondents’ personal rating of the keyword “psychiatric” (Questions Nr. 1 to 2a). Possible structural problems in emergency medical care were also addressed here; (ii) in the second part, a typical case from every day’s work of an EM was presented on the basis of a case vignette in order to determine the emergency medical procedure and possible problem-solving strategies (Questions Nr. 3 to 5); (iii) the third part of the questionnaire dealed with the need for improved training and continuing education opportunities relating to clinical psychiatric conditions (Questions Nr. 6 to 7). Demographic data were collected at the end of the questionnaire. The questionnaires were distributed by contact persons in the respective hospitals and returned using the return boxes provided or directly to the authors. Prerequisites for participation in the survey were active participation in emergency medical services or regular shifts in a ward with acute psychiatric admissions.

### Analysis of the questionnaires

Responses were recorded using Microsoft EXCEL 2021® (Microsoft Corp., Redmond, WA). The statistical analyses were performed with Sigma Plot Version 14® for Windows (Systat Software GmbH, Erkrath, Germany) and SPSS Version 28® (Statistical Package for Social Science, IBM, Armonk-New York, USA).

The descriptive analysis of the questionnaire characteristics surveyed differed depending on the variable type and was performed by means of frequencies and percentages in the case of categorical characteristics or using arithmetic mean, standard deviation (SD), median, and range in the case of metric scaled characteristics. Further evaluation of possible differences between the two groups of emergency medicine and psychiatry physicians was performed by means of suitable exploratory hypothesis tests, for which a two-sided type 1 error rate of 5% was generally assumed. Here, the chi-square test was used for multiple response sets in the case of categorical end-points. Metrically scaled end-points were evaluated using either the unpaired t-test or the Mann-Whitney U test after checking the normal distribution assumption via the Shapiro-Wilk test.

## Results

**Fig. 1:**
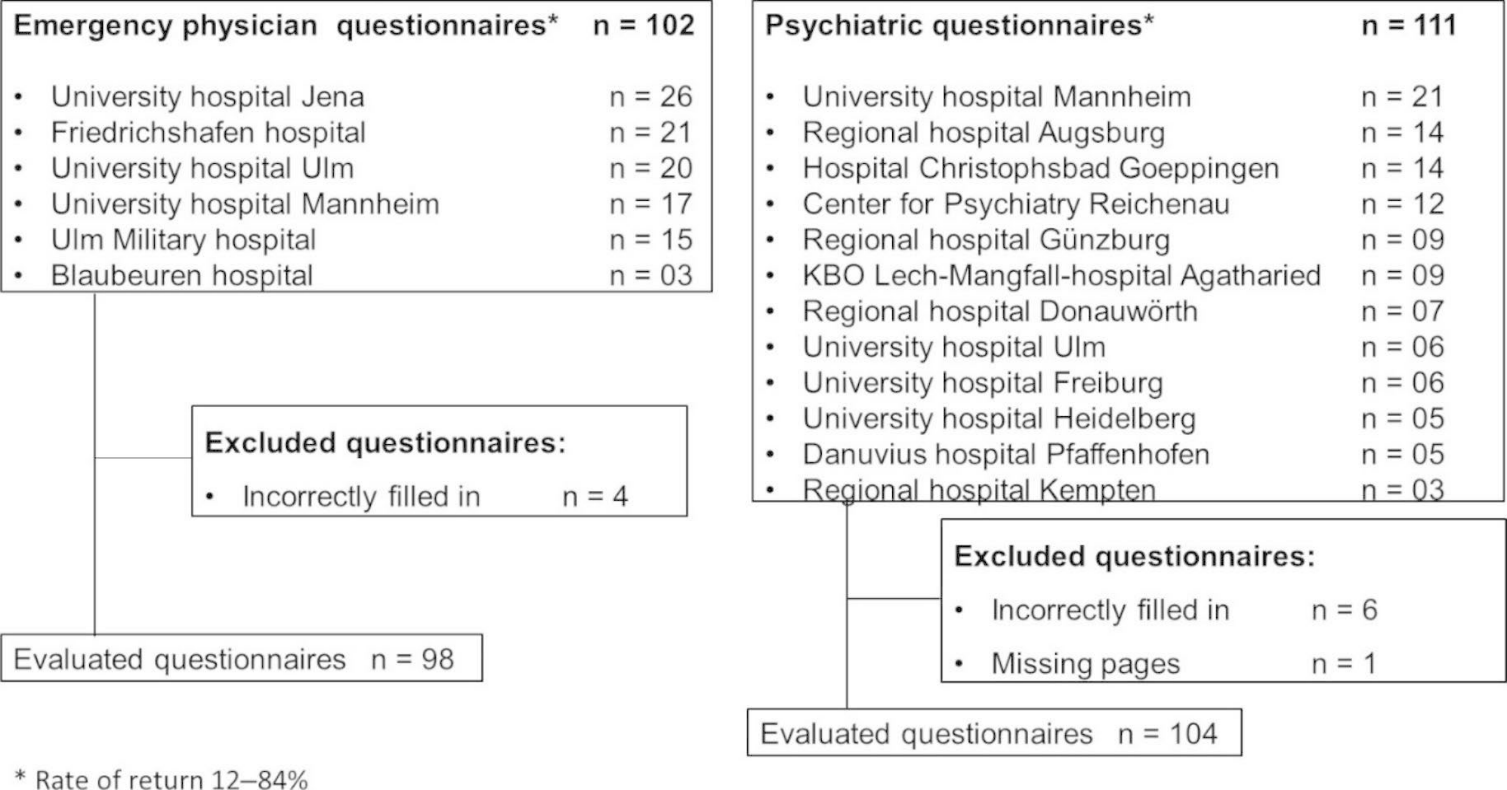
Flowchart of the study

Figure 1 provides an overview of the participating clinics, differentiated by emergency physician location and psychiatric treatment facilities. The response rate of the questionnaires varied between 12 and 84%. The reason for this was that 20 questionnaires were initially handed out to each clinic.

Among the emergency physician locations surveyed, the questionnaire return rate was greater than 90% for 5 of 6 of the respondents. One site had a return rate of only 15%. The reason for this is unknown, since survey responses were anonymous. Among the psychiatric hospitals surveyed, the return rate was less homogeneous, as shown in the flowchart in Fig. 1. One hospital had a return rate of 100%. The return rate of the other hospitals was as follows: 2 hospitals with 70%, 1 hospital with 60%, 2 hospitals with 45%, 1 hospital with 35%, 2 hospitals with 30%, 2 hospitals with 25%, and 1 hospital with 15%. The translated questionnaires can be found in the Supplement (questionnaire EM, questionnaire PS).

### Results for Questions 1 and 2 (operational keyword ”causes of rejection” of psychiatric emergency patients in acute psychiatry)

Statistical analyses using chi-square tests for multiple response sets revealed a global p-value of < 0.001 between EM and PS in terms of significant differences in response options (see Table 1). Specifically, as shown in Tables 1 and 28.6% of EM indicated that their general therapeutic motivation was lower when responding to an emergency with a psychiatric indication than when other indications were present. Among PS, 42.3% indicated that they often perceive the emergency physician’s admission indications as erroneous and therefore have lower motivation when they encounter psychiatric emergency patients. Significantly more EM (36.7%) than PS (11.5%) reported that they did not feel sufficiently qualified to handle emergencies involving psychiatric patients. At 19.2%, a statistically significant relevant proportion of PS felt anxious about a possible EM referral of an acute psychiatric patient. 46.9% of EM and 56.7% of PS reported that they did not differentiate between emergency psychiatric patients and other emergencies.

**Table 1:** Results for Questions 1 and 2

	Emergency Physician n = 98	Psychiatrist n = 104	p-Value
**Question 1 – Rating of the emergency call “psychiatric emergency"**			
*EM: As an emergency physician, you read the text " Emergency – psychiatric” on your pager.* *What does that trigger in you?* *PS: You are informed by your nurse that the emergency physician is on the phone and wants to admit a patient. What does this trigger in you?*			
Rating of the psychiatric emergency as “meaningless” or as “incorrect diagnosis, made by the EM”	28 (28.6%)	44 (42.3%)	0.1770
Feeling of insufficient qualification for psychiatric emergencies	36 (36.7%)	12 (11.5%)	0.0001
Anxious about the psychiatric emergency	3 (19.2%)	20 (3.1%)	0.0010
Making no difference between psychiatric emergencies and other emergencies	46 (46.9%)	59 (56,7%)	0.6344
**Question 2 - Reasons for refusal of hospital admission**			
*EM: Have you ever had the problem that you, as an emergency physician, indicated a patient for admission to psychiatry, but the on-site psychiatry department had to decline admission? What was the reason?* *PS: Have you ever had the problem that you, as a psychiatrist, had to reject an admission when the emergency physician indicated that the patient should be admitted to the psychiatric unit? What was the reason?*			
**Question answered “Yes”**	**Emergency Physician** **n = 80**	**Psychiatrist** **n = 85**	
No capacity	29 (36.3%)	20 (23.5%)	0.1683
Intoxication of the patient	63 (78.8%)	75 (88.2%)	0.2005
Preclinically applied medication	43 (53.8%)	54 (63.5%)	0.1956
Patient not assigned to the hospitals catchment area	55 (68.8%)	41 (48.2%)	0.0865

Table 1 summarizes the results of the questions one and two (see Supplement). The questions from the questionnaires are shown as examples before the summarized response items of each question. The responses of emergency physicians and psychiatrists are presented in absolute values as well as percentages. Statistical differences were calculated by means of pairwise chi-square tests. EM = Emergency Physician, PS = Psychiatrist

More than 80% of the EM and PS interviewed had already been in a situation where a psychiatric emergency patient was rejected or had to be rejected by the psychiatric department (emergency physicians n = 80, 81.6%, psychiatrists: n = 85, 81.7%). Various reasons for refusal of an acute psychiatric patient were reported by EM and PS. As shown in Table 1, acute shortage of capacity (no free beds) was reported equally by EM and PS (EM n = 29, 36.3%, PS n = 20, 23.5%). The most common reason for refusal was intoxication (EM n = 63, 78.8%, PS n = 75, 88.2%). Correspondingly, EM and PS stated that emergency medical therapy had been the reason for rejection by acute psychiatry (EM n = 43, 53.8%, PS n = 54, 63.5%). The second most common reason given by the emergency physicians was the fact that the patient did not come from the catchment area of the hospital (EM n = 55, 68.8%, PS n = 41, 48.2%). The term “catchment area” is defined as the geographically assigned area in which the psychiatric hospital is located and, as result, the people the psychiatric hospital serves. If a patient comes from another area, outside of the catchment area, a psychiatric emergency may not be admitted simply because the patient is from another geographic location. This, in turn, leads to conflicts between emergency physicians and psychiatrists (or psychiatric hospitals).

### Results of questions 3–5 – casuistry, emergency plan

A casuistry based on a scenario frequently encountered in daily emergency medical practice was presented in Questions 3 to 5. EM and PS were asked about their assessment of the situation (Question 3), possible therapeutic strategies (Questions 3 and 3a), and their opinion on the practical relevance of a tailored, case-specific treatment protocol (Questions 4 and 5). In Question 3 (casuistry: post-traumatic stress disorder, agitation, hyperventilation, no verbal calming possible), EM were asked how they would proceed further. Physicians working in psychiatric care were asked to describe how they would proceed in the situation described based on their expertise.

**Table 2:** Results of the Questions 3 to 4

Variable	Emergency Physician N = 98	Psychiatrist N = 104	
**Question 3 - Casuistry: post-traumatic stress disorder, agitation, hyperventilation, no verbal calming possible**			
*EM: Please imagine the following scenario: You are called as an emergency physician to a patient with post-traumatic stress disorder. When you arrive, the patient is agitated, hyperventilating and cannot be calmed down verbally. How do you proceed?* *PS: Please imagine the following scenario: An emergency physician visits a patient with known post-traumatic stress disorder. Upon the arrival of the emergency physician, the patient is agitated, hyperventilating, and cannot be calmed verbally. What would you do, based on your psychiatric expertise?*			
• Talk down technique	90 (92.1%)	95 (91.3%)	0.4434
• Benzodiazepine administration	69 (70.4%)	82 (78.8%)	0.1272
• Hypnotic administration (e.g. propofol)	6 (6.1%)	0 (0.0%)	0.0155
• Antipsychotic administration (e.g. haloperidol)	2 (2.0%)	5 (4.8%)	0.2342
• Involving the police to obtain psychiatric admission	7 (7.1%)	4 (3.8%)	0.3926
• Seek phone contact with the acute psychiatric hospital	83 (84.7%)	55 (52.9%)	0.0107
• Abandoning all further attempts to ensure admission	0 (0.0%)	7 (6.7%)	0.0067
**Question 3a - Different options of medication application in the Casuistry/psychiatric emergency**			
*EM and PS: Which type of medication application would you prefer in such a case?*			
Intra venous	38 (38.8%)	4 (3.8%)	0.0001
Intra osseous	0 (0.0%)	0 (0.0%)	n.e.
Mucosal Atomization Device	36 (36.7%)	11 (10.6%)	0.0006
Intra muscular	5 (5.1%)	9 (8.7%)	0.1624
Oral drug administration	66 (67.3%)	97 (93.3%)	0.0001
None of the options mentioned above	2 (2.0%)	5 (4.8%)	0.1742
**Question 4 - Possible items of a treatment protocol for the psychiatric emergency**			
*EM: Imagine the following alternative: In the situation described above, you contact the psychiatrist by telephone. The psychiatrist tells you that a treatment concept for acute situations has been drawn up for the patient. Upon request, the patient’s mother hands over the document to you. As an emergency physician, what information would you like to be included on such a treatment protocol?* *PS: Imagine the following alternative: In the situation mentioned above, a treatment protocol for acute* *Situations has been prepared for the patient, which the patient’s mother hands over to the emergency physician. As a psychiatrist, what information would you want to communicate to the emergency physician by means of such a treatment protocol?*			
Possible symptoms of the dissociative seizure	81 (82.7%)	78 (75.0%)	0.9817
The expected duration of the seizure	66 (67.3%)	62 (59.6%)	0.8977
Helpful behavior	91 (92.3%)	100 (96.2%)	0.3043
The point at which medical therapy should be considered	73 (74.5%)	67 (64.4%)	0.7778
The type and dosage of the drug to be used	87 (88.8%)	79 (76.0%)	0.6995
Circumstances under which outpatient care would be possible	86 (87.8%)	69 (66.3%)	0.2318
Conditions for inpatient admission	87 (88.8%)	91 (87.5%)	0.5312
Question cannot be answered	0 (0.0%)	2 (1.9%)	0.1485

Table 2 summarizes the results of the questions three to four (see Supplement). The questions from the questionnaires are shown as examples before the summarized response items of each question. The responses of emergency physicians and psychiatrists are presented in absolute values as well as percentages. Statistical differences were calculated by means of pairwise chi-square tests. EM = Emergency Physician, PS = Psychiatrist, n.e. = not estimated

Again, multiple set analysis revealed a global, statistically significant difference between the responses (p < 0.001).

As shown in Table 2, both EM and PS would most frequently choose the “talk down” technique as an appropriate approach (EM n = 90, 92.1%, PS n = 95, 91.3%). Both survey groups were also equally likely to administer benzodiazepines (EM n = 69, 70.4%, PS n = 82, 78.8%). Although a statistically significant number of EMs (6.1%) would also consider a hypnotic such as propofol, this was not an option for any of the PSs interviewed. Only a few of the respondents would administer an antipsychotic (EM n = 2, 2.0%, PS n = 5, 4.8%). Involving the police to obtain psychiatric admission would only be considered by a small percentage of respondents (EM n = 7, 7.1%, PS n = 4, 3.8%). Significantly more EM than PS would seek phone contact with the acute psychiatric hospital (EM n = 83, 84.7%, PS n = 55, 52.9%). Abandoning all further attempts to ensure admission of the patient would be an option for statistically significant more PSs (EM n = 0, 0.0%, PS n = 7, 6.7%).

In Question 3a (see Table 2), EM and PS were asked about the preferred mode of application of a drug, related to the casuistry from Question 3.

Statistically more EM than PS would opt for intravenous drug administration (EM n = 38, 38.8%, PS n = 04, 3.8%), while intraosseous drug administration would not be a primary consideration for any of the respondents. EM were statistically significant more likely to choose a MAD (Mucosal Atomization Device) (EM n = 36, 36.7%, PS n = 11, 10.6%). Intramuscular drug administration would be selected by a small proportion of respondents (EM n = 5, 5.1%, PS n = 9, 8.7%). Oral drug administration would be the most common choice for the physicians surveyed (EM n = 66, 67.3%, PS n = 97, 93.3%). Finally, 2 EM (2.0%) and 5 PS (4.8%) stated that they would not choose any of the application methods listed.

In Question 4, EM and PS were asked to imagine that an emergency plan prepared by psychiatric specialists was available for the patient in the casuistry presented in Question 3. The following information was rated by respondents as useful or not useful (see Table 2): Possible symptoms of the dissociative seizure were rated as useful information by almost the same number of respondents in each group (EM n = 81, 82.7%, PS n = 78, 75%). The expected duration of the seizure was rated as useful by more than half of physicians (EM n = 66, 67.3%, PS n = 62, 59.6%). Helpful behavior such as pointing out the effect of verbal reassurance or the presence of a special caregiver was rated as useful by almost all respondents (EM n = 91, 92.3%, PS n = 100, 96.2%). The point at which medical therapy should be considered was still rated as useful by more than half of respondents (EM n = 73, 74.5%, PS n = 67, 64.4%). The type and dosage of the drug to be used are rated as important information by a similar number of physicians in each group (EM n = 87, 88.8%, PS n = 79, 76.0%). EM would more often want to know the circumstances under which outpatient care would be possible (EM n = 86, 87.8%, PS n = 69, 66.3%). Regarding the conditions for inpatient admission, respondents agreed that this information could be important for the emergency physician (EM n = 87, 88.8%, PS n = 91, 87.5%). In Question 5, physicians were asked to rate the concept of an emergency plan as relevant or not relevant in practice. Both groups of physicians rated the concept as relevant in practice (EM n = 91, 92.9%, PS n = 96, 93.2%).

### Results of questions 6–7a

Question 6 asked both EM and PS about their need for continuing education and training on the topic of psychiatric emergencies or for a better insight into emergency medicine, in the form of a one-day internship. About the same number of respondents in each group, 78.6% versus 74% (χ²(1) = 0.21, p = 0.647), expressed a personal need for this education. Question 7 asked PS if they saw a need for additional training on psychiatric emergencies for EM, with 97 PS (93.3%) answering “yes.” In Question 7, the EM who saw a need for further training were asked to name specific training topics. The physicians were asked to rank their need qualitatively from extremely important to not at all important. Similarly, in Question 7a, the PS were asked to rate the relevance of the training topics for EM. Both EM and PS rated the topics “dissociative seizure,“ “intoxication,“ and “legal aspects of psychiatric emergencies” as significantly important education topics (Mann-Whitney U test, p < 0.001). Suicidality was identified as an extremely important training topic by more than half of the emergency physicians (53.2%) and 69.1% of the psychiatrists (Mann-Whitney U test, p = 0.073). The physicians surveyed rated dealing with self-aggression and aggression towards others as not important (p > 0.05) (see Supplement, Table 1).

## Discussion

For the first time, the results of the present study distinctly report the problems affecting interaction between relevant care facilities in the emergency medical care of psychiatric patients from the perspective of EM and PS. Major problems are caused by differences in the assessment of psychiatric emergencies by EM and PS. In particular, pre-clinical drug therapy and assessment of the severity of intoxication were identified as problems affecting interaction. In the case described in our questionnaire, the emergency physician’s approach was congruent with the approach of experienced PS in its essential points. Surprisingly, however, significantly more EM than psychiatrists would seek contact with psychiatry hospitals. The need for further training for EM with regard to psychiatric syndromes was rated as important by both groups surveyed. However, 2/3 of the PS interviewed would also like to have better insights into the area of emergency medicine.

### Problems affecting interaction between those administering emergency medical therapy and those providing further psychiatric treatment

The reporting pattern of “psychiatric emergency” evokes negative associations on both sides of the chain of caregiving. However, the reasons for this are different in each specialty. Similar to the findings reported by Pajonk et al. (1998), a not inconsiderable proportion of emergency physicians in the present survey also feel inadequately qualified to deal with psychiatric emergencies [10]. For the respondent psychiatrists, a frequently reported point of criticism was that, from their perspective, the emergency physician’s admission indication for the psychiatric emergency was either incomprehensible or was assessed as erroneous. By contrast, studies among EM and U.S. paramedics show that the diagnostic quality for psychiatric emergencies is consistently high compared to that for somatic illnesses. [9, 18]. However, the crucial factor with regard to the negative connotation of the term psychiatric emergency seems to be based in the problems associated with patient care. In particular, the fact that the EM interviewed are commonly faced with a refusal to admit the patient to the acute psychiatric department seems to be a burdening factor in the cooperation between the disciplines involved. Concordantly, the PS interviewed report that they have had to refuse to admit patients referred by EM in the past. In acute medicine, there is a discernible trend that is seeing an increasing number of hospitals and probably also psychiatric admission wards signing off with the coordinating rescue coordination centers for capacity reasons [19]. The frequency of rejection of psychiatric emergency patients and the underlying causes have not yet been investigated systematically either for Germany alone or for the European Union as a whole. Intoxication was identified as the most common reason for rejection in the present study. In this context, respondents further indicated that the emergency medical therapy, i.e., prehospital medication, would often also be cited as a reason preventing admission. In practice, the assessment of the severity of intoxication is always a point of contention between the disciplines surveyed. Naturally, an emergency physician with anesthesiological expertise will evaluate a patient with reduced vigilance due to sedation and who is possibly also intoxicated in a different way than a physician working in the field of psychiatry without experience in airway or circulation management. In the absence of data regarding monitoring after emergency medical therapy with hypnotics/benzodiazepines, it is worthwhile to look at the anesthesiology guidelines for monitoring patients after diagnostic and therapeutic measures [20, 21]. The minimum requirements regarding monitoring (pulse oximetry +/- electrocardiogram, non-invasive blood pressure measurement, capnometry) are usually not met in an acute psychiatric setting. Likewise, the personnel requirements for safe monitoring are not fulfilled [20, 21]. Therefore, the observation of such patients in an emergency room as demanded by many psychiatrists is understandable – not least in order to accomplish what is referred to as “medical clearance,“ i.e., the exclusion of organic causes behind the psychiatric symptoms. [22–24]. One option for monitoring and treating psychiatric emergency patients is offered by what are known as “Psychiatric Intensive Care Units (PICUs),“ which are available in a small number of hospitals in Europe. These units have specially trained psychiatric staff, the necessary equipment for monitoring and therapy, and can fall back on an intraclinical, multidisciplinary emergency backup in case of an emergency. [25–27].

In order to avoid the problem of the discrepancy between emergency medical therapy and the lack of psychiatric monitoring resources, a procedure should be adopted at the emergency scene that does not a priori make further treatment in an acute psychiatric hospital impossible.

### Case vignette: emergency plan for prehospital care of psychiatric patients

In the case presented, EM and PS agreed on their approach to treating a patient in an acute stressful situation. Both verbal calming of the situation by means of the “talk-down technique” and “rapid tranquilization” by administering benzodiazepines are techniques reported in the literature and rated by interviewees as adequate in the acute setting [28–30]. The significant number of EM who would administer benzodiazepine to a patient in an acute state of agitation contradicts the results of Tonn et al. (2004), whose analysis of the medication administered for psychiatric indications showed that EM only very rarely resort to administering benzodiazepine and even more rarely to neuroleptics [31]. If medication administration was considered, EM would be significantly more likely than psychiatrists to use intravenous administration or a mucosal atomization device (MAD) as alternative methods of administration in addition to oral medication administration. For patients in acute states of agitation, it is difficult to imagine that intravenous administration in particular would be feasible without coercion. Although MAD systems are routinely and safely used in pediatric (pre)medication, there are no systematic studies for this type of drug application in adults experiencing acute states of agitation [32]. In addition to medicinal therapy, there are other support systems in place to assist patients and the emergency physicians who attend them. Emergency chaplains, for example, can accompany patients when desired, and they have the potential to reduce the workload of the emergency physician. Yet, chaplains are not able to replace the emergency physician, due to an ethical/legal dilemma. Every emergency physician must be able to rule out the potential that the patient may harm themself or a third-party. If there is any uncertainty about safety in the pre-clinical setting, protocol requires a specialist psychiatric assessment in a psychiatric hospital. Probably the most effective approach to avoiding interface problems would be to establish phone contact with an experienced PS [33, 34]. Significantly more EM would seek this contact than their psychiatric counterparts. Because many psychiatric disorders are chronic, the likelihood that these patients will require emergency medical care at irregular intervals is higher compared to the general population [3, 4]. Indeed, these patients are sometimes also called “revolving door patients.” In the absence of an organic cause, however, a concept that prevents recurrent inpatient admissions should be available for these patients in particular. For this reason, the authors of this study presented a concept to the physicians interviewed, which defines the procedure in the acute situation in the sense of an “emergency plan.” The concept was evaluated positively in principle. From an emergency physician’s perspective, however, the desire for such an emergency plan also emphasizes the physician’s own uncertainty with regard to the management of psychiatric emergencies. No data are yet available with respect to the practicability of a tailored patient plan of this kind.

Most respondents perceive a considerable need for continuing education for EM regarding the care of psychiatric emergency patients. This is in line with the results of other studies and ultimately reflects the need for improved training and further education of emergency physicians in line with demand [10, 12, 15, 29].

Not only is training in pre-clinical psychiatric emergency response inadequate for emergency physicians, but Pajonk and colleagues recognized the training deficit more than 20 years ago [10]. The curriculum for emergency physicians in Germany should include a more intensive preparation for psychiatric emergencies, which would be a first step towards improving the emergency medical care of psychiatric patients. Also, the fact that over 90% of the psychiatrists surveyed in this study called for better training of emergency physicians regarding pre-clinical psychiatric emergencies reiterates that there is a significant training problem. Emergency physicians cannot obtain a detailed psychopathological assessment without adequate training, and they are also limited by the restrictive time schedules in emergency medicine. These restraints have led some to question whether symptom-based therapy may not be sufficient for pre-clinical care of psychiatric emergencies. Others wonder whether it would be possible to bring a psychiatrist trained in emergency medicine to the patient in the event of a psychiatric emergency. There are already pilot projects in Germany in which, within the framework of so-called home treatment and intensified outpatient therapy, the psychiatrist provides care for the patient at home after an inpatient stay [36]. However, such models have not yet been established for psychiatric emergencies. As of yet, it is not possible for a psychiatrist to act as an emergency physician in Germany, due to the underlying juridical conditions (experience in intensive care medicine, intubation experience, etc.). Taking into account the results of the present study, admission criteria were defined to guide EM with regard to the inclusion and exclusion criteria for direct referral of the psychiatric emergency to an acute psychiatric hospital (see Supplementary Fig. 1). The proposed admission criteria are adapted from the German “Emergency Psychiatry” guideline [14]. The criteria can guide hospitals as they develop their own criteria for the admission of psychiatric emergencies, taking into account the personnel and equipment available to them. This in turn should help to minimize the problems identified that affect interaction between the two settings.

### Study limitations

Although the present study is based on a multicenter approach, it can nevertheless only be applied to other, especially rural, emergency physician locations to a limited extent. General practitioners and other physicians not primarily involved in emergency medicine typically provide emergency care in rural areas in Germany. However, the institutions that participated in this study were almost exclusively anesthesiologist-oriented, hospital-associated emergency physician locations. It was apparent in the run-up to the study that the topic of “psychiatric emergency” is fraught with many negative emotions on both sides. In view of this, the possibility of a response bias in the sense of an error of extreme tendency cannot be excluded. The accompanying free text responses, some of which were very harsh, suggest that this bias is indeed in play, but these responses were not taken into account in the final evaluation for statistical reasons. The content design of the questionnaire with successive questions can naturally result in a question series effect. For reasons of anonymity, no further details such as the exact age or sex of the participating physicians were requested.

## Conclusion

Problems affecting interaction with other departments or facilities in the emergency medical care of psychiatric patients relate, on the one hand, to structural problems that cannot be modified, such as a lack of admission capacity and non-existent or inadequate monitoring facilities at acute psychiatric hospitals. On the other hand, some factors, such as the training of EM, but also communication between EM and PS, can be improved. Tailored approaches, such as the development of emergency plans for patients who are in frequent contact with prehospital emergency medicine departments, could help to optimize patient-centered emergency care in a way that is accepted by both EM and PS.

### Electronic supplementary material

Below is the link to the electronic supplementary material.

Supplementary Material 1

## Data Availability

The datasets generated and/or analyzed as part of this study are not publicly available because the further comments added as free were a priori not intended for publication. They are primarily used for internal quality analyses. All other results and data collected are reported entirely within the scope of this manuscript. If there is a justified interest, the data can be requested from the corresponding author.

## Abbreviations

EP: Emergency Physician
MAD: Mucosal Atomization Device
PS: Psychiatrist
PICU: Psychiatric Intensive Care Unit
WHO: World Health Organization
