# Correction: Emergency medical care of patients with psychiatric disorders - challenges and opportunities: Results of a multicenter survey

**DOI:** 10.1186/s12873-024-01045-3

**Published:** 2024-07-23

**Authors:** Benedikt Schick, Benjamin Mayer, Markus Jäger, Bettina Jungwirth, Eberhard Barth, Martin Eble, Christoph Sponholz, Claus-Martin Muth, Carlos Schönfeldt-Lecuona

**Affiliations:** 1 Department of Anesthesiology and Intensive Care Medicine, University Hospital Ulm, Albert-Einstein- Allee 23, 89081 Ulm, Germany; 2 Institute of Epidemiology and Medical Biometry, Ulm University, Schwabstraße 13, 89075 Ulm, Germany; 3 Department of Psychiatry, Psychotherapy and Psychosomatic, District Hospital Kempten, Kempten, Germany; 4 Department of Anesthesiology, Intensive Care Medicine, Emergency Medicine & Pain Therapy, Klinikum Friedrichshafen GmbH, Röntgenstraße 2, 88048 Friedrichshafen, Germany; 5 Department of Anesthesiology and Intensive Care Medicine, Jena University Hospital, Friedrich Schiller University Jena, Am Klinikum 1, 07747 Jena, Germany; 6 Department of Psychiatry and Psychotherapy III, University Hospital Ulm, Leimgrubenweg 12–14, 89075 Ulm, Germany

**Correction: BMC Emergency Medicine (2022) 22:173**

10.1186/s12873-022-00722-5

The original article contains an error in Table 1 whereby the two percentages reported in the ‘Anxious about the psychiatric emergency’ row are erroneously inverted.

The ‘Emergency Physicians’ column should instead state ‘3 (3.1%)’ and the ‘Psychiatrists’ column should instead state ‘20 (19.2%)’.

