# Supplementary material for: Emergency medical care of patients with psychiatric disorders - challenges and opportunities: Results of a multicenter survey
Source: BMC Emerg Med. 2022 Oct 28;22:173. doi: 10.1186/s12873-022-00722-5 (PMC9615220; doi:10.1186/s12873-022-00722-5)
Supplement: Supplementary file 1 — Supplementary Material 1 [file 12873_2022_722_MOESM1_ESM.docx]

**Emergency medical care of patients with psychiatric disorders: challenges and opportunities. Results of a multicenter survey**

Benedikt Schick🖂, Benjamin Mayer, Markus Jäger, Bettina Jungwirth, Eberhard Barth, Martin Eble, Christoph Sponholz, Claus-Martin Muth, Carlos Schönfeldt-Lecuona.

**Supplement**

**Table 1 – Overview of answers to Questions 6–7a**

| **Question** | **Emergency Physicians** | **Psychiatrists** |
| --- | --- | --- |
| **Question 6 – Individual emergency concept as a reasonable option for emergency physicians** | | |
| Yes | 91 (92.9%) | 96 (92.3%) |
| No | 7 (7.1%) | 7 (7.7%) |
| **Question 7 – Better insight into psychiatry or emergency medicine** | | |
| Yes | 77 (78.6%) | 77 (74.0%) |
| No | 21 (21.4%) | 27 (26.0%) |
| **Question 7a – More advanced training on psychiatric emergencies for emergency physicians (from the perspective of psychiatrists)** | | |
| Yes |  | 97 |
| No |  | 7 |

**
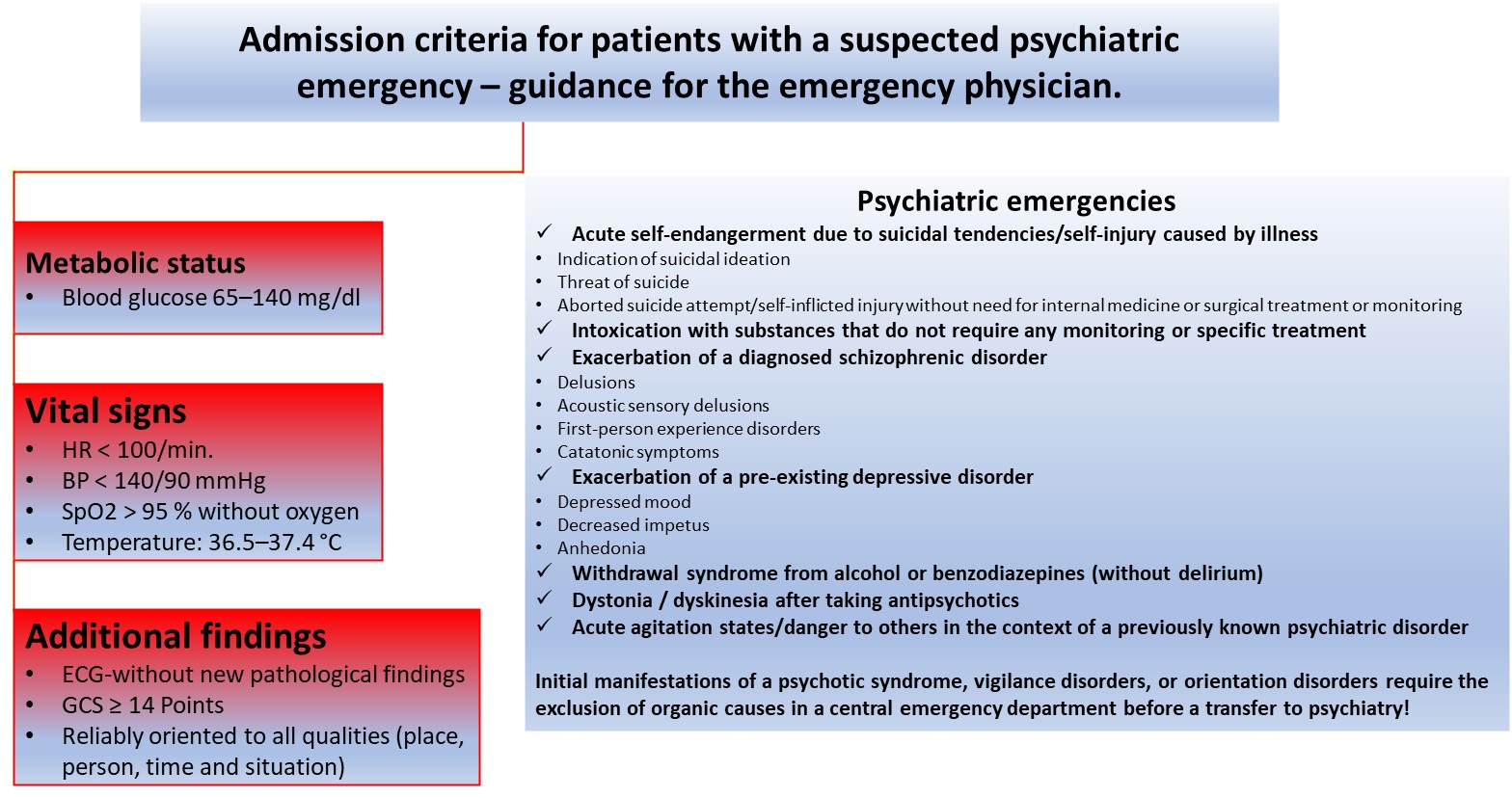
Figure 1 Supplement – Possible admission criteria for psychiatric emergencies in acute psychiatry – overview for emergency physicians.**

HR: Heart Rate, BP: Blood Pressure, ECG: Electrocardiogram, GCS: Glasgow Coma Scale,

**Questionnaire for emergency physicians**

**1.**  As an emergency physician, you read the text " Emergency – psychiatric" on your pager.

What does that trigger in you? (Multiple answers possible)

|  | Sometimes I perceive psychiatric emergencies as pointless, so my intrinsic motivation can be quite low in such emergencies. |
| --- | --- |
|  | I frequently have the feeling that I am actually not sufficiently qualified to treat psychiatric patients. |
|  | I'm scared because I don't know what to expect in the situation. |
|  | I rate it as "neutral;" it's just an emergency like any other. |

You may also add further comments as free text.

**2.** Have you ever had the problem that you, as an emergency physician, indicated a patient for admission to psychiatry, but the on-site psychiatry department had to decline admission?

|  | Yes |  |  | No |
| --- | --- | --- | --- | --- |

**2 a.** If yes, what was the reason (multiple answers possible)?

|  | No available bed. |
| --- | --- |
|  | Patient is intoxicated and must first be monitored in the emergency room. |
|  | Emergency medical therapy (sedation/antipsychotics) impedes admission because the patient may need to be monitored closely. |
|  | Patient is not from the local psychiatric hospital's catchment area. |

You may also add further comments as free text

**3.** Please imagine the following scenario:

You are called as an emergency physician to a patient with post-traumatic stress disorder. When you arrive, the patient is agitated, hyperventilating and cannot be calmed down verbally. How do you proceed? (Multiple answers possible)

|  | I try to calm the patient verbally using the "talk down" approach. |
| --- | --- |
|  | I administer a benzodiazepine (e.g., midazolam/lorazepam) to the patient to calm them down. |
|  | I sedate the patient with a hypnotic (e.g., propofol) to calm them down. |
|  | I administer an antipsychotic (haloperidol) to calm the patient. |
|  | I ask the police for help in obtaining admission to the psychiatric ward. |
|  | I call the psychiatric department where the patient is already known and try to find a solution with my colleagues there over the phone. |
|  | In the end, I explain to the parents and friends present that the patient’s condition is improving on its own and, after consulting with the psychiatric department, I cancel the intervention. |

You may also specify further options that you consider suitable as free text.

**3 a.** Which type of medication application would you prefer in such a case?

|  | Intravenous administration. |
| --- | --- |
|  | Intraosseous administration. |
|  | MAD system (Mucosal Atomization Device) – "nasal atomizer." |
|  | Intramuscular administration. |
|  | Oral administration (if possible). |
|  | None of the above. |

**4.** Imagine the following alternative:

In the situation described above, you contact the psychiatrist by telephone. The psychiatrist tells you that a treatment concept for acute situations has been drawn up for the patient. Upon request, the patient's mother hands over the document to you. As an emergency physician, what information would you like to be included on such a treatment protocol? (Multiple answers possible)

|  | Possible symptoms (e.g., self-aggressive or aggressive towards others, screaming, crying, etc.) and expected progression. |
| --- | --- |
|  | Expected duration of the psychological emergency situation. |
|  | Supportive behavior (verbal reassurance, special caregiver, etc.). |
|  | Point at which medical intervention should be considered. |
|  | Type of medication and dosage that is usually sufficient – placebo use if appropriate, if previously agreed with the patient. |
|  | Outpatient care possible if the following criteria are met.... |
|  | Inpatient admission recommended if the following criteria are met.... |
|  | I cannot answer this question. |

You may also specify further options that you consider suitable as free text

**5.** Do you think a concept such as that outlined above would be appropriate in practice?

|  | Yes |  |  | No |
| --- | --- | --- | --- | --- |

**6.** Would you like more training on psychiatric emergencies for your emergency medicine practice, for example in the form of a one-day internship in an acute psychiatry setting?

|  | Yes |  |  | No |
| --- | --- | --- | --- | --- |

**7.** If yes, which topics would you consider particularly important in this context?

|  | extremely important | very important | relatively important | somewhat important | not at all important |
| --- | --- | --- | --- | --- | --- |
| Psychogenic seizure |  |  |  |  |  |
| Dealing with self-aggression and aggression towards others |  |  |  |  |  |
| Suicidality |  |  |  |  |  |
| Intoxication |  |  |  |  |  |
| Legal aspects of dealing with psychiatric patients |  |  |  |  |  |

You may also specify other important topics as free text.

In the questions below, we ask you to provide some information about yourself. The information you provide will not allow any conclusions to be drawn about your person.

**8.** How old are you?

|  | < 25 years |
| --- | --- |
|  | 25–35 years |
|  | 35–45 years |
|  | 45–55 years |
|  | 55–65 years |
|  | > 65 years |

**9.** Which medical discipline do you belong to (intended residency or specialist qualification)? (Multiple answers possible)

|  | Anesthesia |
| --- | --- |
|  | Internal medicine |
|  | Psychiatry |
|  | Neurology |
|  | Other discipline |

**10.** How many years have you held the additional designation of emergency medicine?

|  | < 1 year |
| --- | --- |
|  | 1–5 years |
|  | > 5 years |
|  | I do not have an additional qualification in emergency medicine |

**Questionnaire for psychiatrists**

**1.** You are informed by your nurse that the emergency physician is on the phone and wants to admit a patient. What does this trigger in you? (Multiple answers possible).

|  | Sometimes I feel that the emergency physician's admission indication is flawed, and my motivation to care for patients admitted by emergency physicians is therefore diminished. |
| --- | --- |
|  | I frequently have the feeling that I am actually not sufficiently qualified to treat psychiatric patients. |
|  | I'm scared because I don't know what to expect in the situation. |
|  | I feel neutral about it; it's just a patient like any other. |

You may also add further comments as free text.

**2.** Have you ever had the problem that you, as a psychiatrist, had to reject an admission when the emergency physician indicated that the patient should be admitted to the psychiatric unit?

|  | Yes |  |  | No |
| --- | --- | --- | --- | --- |

**2a.** If yes, what was the reason (Multiple answers possible)

|  | No available bed. |
| --- | --- |
|  | Patient is intoxicated and must first be monitored in the emergency room. |
|  | Emergency medical therapy (sedation/antipsychotics) impedes admission because the patient potentially needs to be monitored closely. |
|  | Patient is not from the local psychiatric hospital's catchment area. |

You may also add further comments as free text

**3.** Please imagine the following scenario:

An emergency physician visits a patient with known post-traumatic stress disorder. Upon the arrival of the emergency physician, the patient is agitated, hyperventilating, and cannot be calmed verbally. What would you do, based on your psychiatric expertise? (Multiple answers possible)

|  | I try to calm the patient verbally using the "talk down" approach. |
| --- | --- |
|  | I administer a benzodiazepine (e.g., midazolam/lorazepam) to the patient to calm them down. |
|  | I sedate the patient with a hypnotic (e.g., propofol) to calm them down. |
|  | I administer an antipsychotic (haloperidol) to calm the patient. |
|  | I ask the police for help in obtaining an admission to the psychiatric ward. |
|  | I call the psychiatric department where the patient is already known and try to find a solution with my colleagues there over the phone. |
|  | In the end, I explain to the parents and friends present that the patient’s condition is improving on its own and, after consulting with the psychiatric department, I cancel the intervention. |

You may also specify further options that you consider suitable as free text.

**3 a.** Which type of medication application would you prefer in such a case?

|  | Intravenous administration. |
| --- | --- |
|  | Intraosseous administration. |
|  | MAD system (Mucosal Atomization Device) – "nasal atomizer." |
|  | Intramuscular administration. |
|  | Oral administration (if possible). |
|  | None of the above. |

**4.** Imagine the following alternative:

In the situation mentioned above, a treatment protocol for acute situations has been prepared for the patient, which the patient's mother hands over to the emergency physician. As a psychiatrist, what information would you want to communicate to the emergency physician by means of such a treatment protocol? (Multiple answers possible)

|  | Possible symptoms (e.g., self-aggressive or aggressive towards others, screaming, crying, etc.) and expected progression. |
| --- | --- |
|  | Expected duration of the psychological emergency situation. |
|  | Supportive behavior (verbal reassurance, special caregiver, etc.). |
|  | Point at which medical intervention should be considered. |
|  | Type of medication and dosage that is usually sufficient – placebo use if appropriate, if previously agreed with the patient. |
|  | Outpatient care possible if the following criteria are met.... |
|  | Inpatient admission recommended if the following criteria are met.... |
|  | I cannot answer this question. |

You may also specify further options that you consider suitable as free text

**5.** Do you think a concept such as that outlined above would be appropriate in practice?

|  | Yes |  |  |  | No |
| --- | --- | --- | --- | --- | --- |

**6.** Do you want a better insight into emergency medical care for your work as a psychiatrist, for example in the format of an optional one-day internship with an emergency medical response vehicle?

|  | Yes |  |  | No |
| --- | --- | --- | --- | --- |

**7.** Do you think that additional training on psychiatric emergencies is needed for colleagues practicing emergency medicine?

|  | Yes |  |  | No |
| --- | --- | --- | --- | --- |

**7a.** If yes, which topics would you consider particularly important in this context?

|  | extremely important | very important | relatively important | somewhat important | not at all important |
| --- | --- | --- | --- | --- | --- |
| Psychogenic seizure |  |  |  |  |  |
| Dealing with self-aggression and aggression towards others |  |  |  |  |  |
| Suicidality |  |  |  |  |  |
| Intoxication |  |  |  |  |  |
| Legal aspects of dealing with psychiatric patients |  |  |  |  |  |

You may also specify other important topics as free text.

In the questions below, we ask you to provide some information about yourself. The information you provide will not allow any conclusions to be drawn about your person.

**8.** How old are you?

|  | < 25 years |
| --- | --- |
|  | 25–35 years |
|  | 35–45 years |
|  | 45–55 years |
|  | 55–65 years |
|  | > 65 years |

**9.** Which medical discipline do you belong to (intended residency or specialist qualification)? (Multiple answers possible)

|  | Anesthesia |
| --- | --- |
|  | Internal medicine |
|  | Psychiatry |
|  | Neurology |
|  | Other discipline |

**10.** How many years have you held the additional designation of emergency medicine?

|  | < 1 year |
| --- | --- |
|  | 1–5 years |
|  | > 5 years |
|  | I do not have an additional qualification in emergency medicine |
